# Diabetic myonecrosis complicated by emphysematous pyomyositis and abscess caused by *Escherichia coli*: a case report

**DOI:** 10.1186/s13256-024-04614-z

**Published:** 2024-07-01

**Authors:** Anne M. Kerola, Kari K. Eklund, Heikki Valleala, Olli Tynninen, Jaakko Helve, Ville Haapamäki, Mari Eriksson

**Affiliations:** 1 Department of Rheumatology, Inflammation Center, Helsinki University Hospital and University of Helsinki, Haartmaninkatu 4, 00290 Helsinki, Finland; 2 Department of Pathology, Helsinki University Hospital and University of Helsinki, Helsinki, Finland; 3 Department of Nephrology, Abdominal Center, Helsinki University Hospital and University of Helsinki, Helsinki, Finland; 4 Radiology, HUS Diagnostic Center, University of Helsinki and Helsinki University Hospital, Helsinki, Finland; 5 Department of Infectious Diseases, Inflammation Center, Helsinki University Hospital, Helsinki, Finland

**Keywords:** Case report, Diabetic myonecrosis, Infectious myositis

## Abstract

**Background:**

Necrotizing myopathies and muscle necrosis can be caused by immune-mediated mechanisms, drugs, ischemia, and infections, and differential diagnosis may be challenging.

**Case presentation:**

We describe a case of diabetic myonecrosis complicated by pyomyositis and abscess caused by *Escherichia coli*. A white woman in her late forties was admitted to the hospital with a 1.5 week history of bilateral swelling, weakness, and mild pain of the lower extremities and inability to walk. She had a history of type 1 diabetes complicated by diabetic retinopathy, neuropathy, nephropathy, and end-stage renal disease. C-reactive protein was 203 mg/l, while creatinine kinase was only mildly elevated to 700 IU/l. Magnetic resonance imaging of her lower limb muscles showed extensive edema, and muscle biopsy was suggestive of necrotizing myopathy with mild inflammation. No myositis-associated or myositis-specific antibodies were detected. Initially, she was suspected to have seronegative immune-mediated necrotizing myopathy, but later her condition was considered to be explained better by diabetic myonecrosis with multifocal involvement. Her symptoms alleviated without any immunosuppressive treatment. After a month, she developed new-onset and more severe symptoms in her right posterior thigh. She was diagnosed with emphysematous urinary tract infection and emphysematous myositis and abscess of the right hamstring muscle. Bacterial cultures of drained pus from abscess and urine were positive for *Escherichia coli*. In addition to abscess drainage, she received two 3–4-week courses of intravenous antibiotics. In the discussion, we compare the symptoms and findings typically found in pyomyositis, immune-mediated necrotizing myopathy, and diabetic myonecrosis (spontaneous ischemic necrosis of skeletal muscle among people with diabetes). All of these diseases may cause muscle weakness and pain, muscle edema in imaging, and muscle necrosis. However, many differences exist in their clinical presentation, imaging, histology, and extramuscular symptoms, which can be useful in determining diagnosis. As pyomyositis often occurs in muscles with pre-existing pathologies, the ischemic muscle has likely served as a favorable breeding ground for the *E. coli* in our case.

**Conclusions:**

Identifying the etiology of necrotizing myopathy is a diagnostic challenge and often requires a multidisciplinary assessment of internists, pathologists, and radiologists. Moreover, the presence of two rare conditions concomitantly is possible in cases with atypical features.

## Background

Necrotizing myopathy is an umbrella term for a broad group of rare diseases, including immune-mediated necrotizing myopathy (IMNM), and necrotizing myopathy caused by drugs, muscle dystrophies, and infections [1]. The differential diagnosis includes diabetic myonecrosis, which is a vaguely understood, rather rare, and underdiagnosed complication of poorly controlled diabetes [2]. Necrotizing myopathies and diabetic myonecrosis represent a diagnostic challenge to neurologists, rheumatologists, and other internists, as defining the cause of muscle necrosis is not always straightforward [1].

We describe a case of diabetic myonecrosis complicated by pyomyositis and abscess caused by *Escherichia coli*, initially suspected to be IMNM. We compare the symptoms and findings typically found in these three entities, aiming to support clinicians with a similar diagnostic challenge.

## Case presentation

A white woman in her late forties presented to the emergency department with a 1.5-week history of pain and stiffness in both of her thighs. Her medical history revealed hypertension and childhood-onset type 1 diabetes complicated by retinopathy, neuropathy, nephropathy, and end-stage renal disease (ESRD). She had no relevant surgical history. Despite her illnesses, she worked full-time in a white-collar job. The pain and stiffness in her thighs had gradually worsened and limited her ability to walk. In the clinical examination, she had reduced muscle strength in her lower extremities, especially in hallux extension and flexion as well as ankle dorsiflexion and plantarflexion. She had no symptoms in her upper limbs or trunk, no joint or respiratory symptoms, and no rash suggestive of dermatomyositis. Her temperature was 37.9 °C, and her laboratory values showed an elevated C-reactive protein (CRP) level of 203 (reference range < 4) mg/l, slightly elevated creatinine kinase (CK) of 700 (reference range 35–210) IU/l, and myoglobin of 1011 (reference range < 50) μg/l. Her urine sample showed significant microscopic hematuria, pyuria, and a positive culture for *Escherichia coli* without any local symptoms of urinary tract infection.

**Fig. 1:**
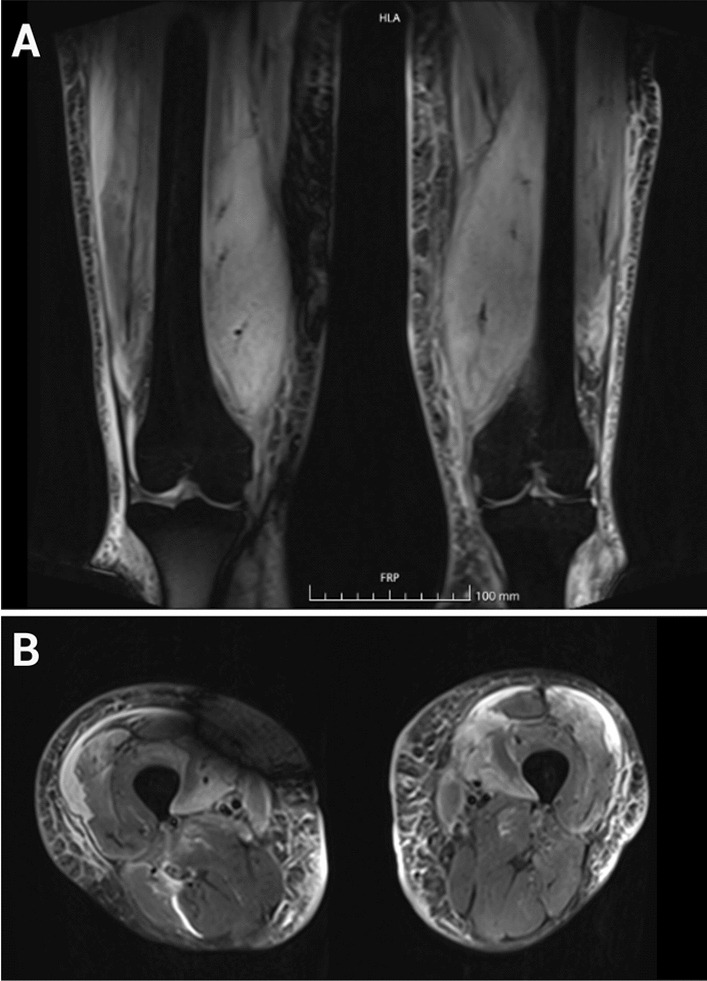
**A**, **B** Extensive bilateral symmetric muscle edema in the quadriceps muscle at initial presentation in an magnetic resonance imaging scan

**Fig. 2:**
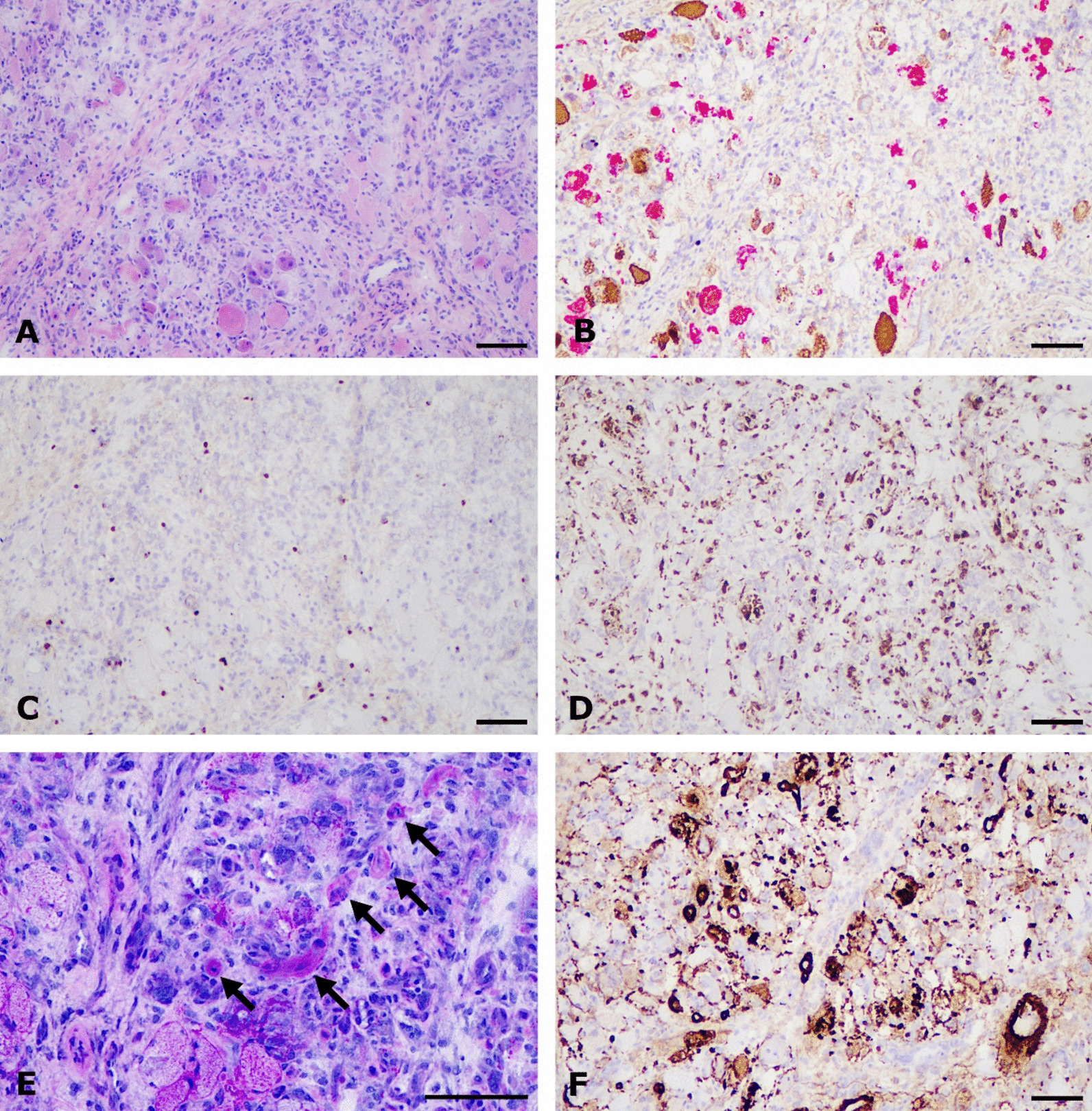
**A** Muscle biopsy shows necrosis, mononuclear inflammatory cells and endomysial fibrosis. Basophilic regenerating muscle fibers and few intact muscle fibers are present (hematoxylin and eosin staining). **B** Myosin heavy chain double staining shows degrading muscle fibers and myophagocytosis (slow fibers diaminobenzidine, fast fibers red). **C** Very mild lymphocytic inflammation in CD3 staining. **D** CD68 staining shows phagocytosis and macrophage infiltration. **E** Hyaline sclerosis of endomysial capillaries (arrows; periodic acid–Schiff staining). **F** Complement C5b-9 membrane attack complex is expressed in necrotic myofibers and in capillaries (scalebar 100 mm).

Her muscle symptoms were examined further by myositis-protocol magnetic resonance imaging (MRI) of her lower limbs, which revealed edema in the muscles of her hips, thighs, shins, and calves, without any significant muscle atrophy or fatty replacement (Fig. 1). Her serology was unremarkable, with no antinuclear antibodies, myositis-associated or myositis-specific antibodies [including anti-3-Hydroxy-3-methylglutaryl-coenzyme A reductase (HMGCR) and anti-signal recognition particle (SRP) antibodies], or signs of viral hepatitis B or C. Muscle biopsy of the left vastus medialis showed extensive necrosis with only mild inflammatory changes (Fig. 2). Muscle fiber regeneration and mild endomysial fibrosis were present. There was strong infiltration of macrophages [cluster of differentiation (CD) 68] and myophagocytosis but only a few T lymphocytes (CD3). Expression of major histocompatibility complex (MHC)-I and C5b-9 membrane attack complex (MAC) were seen in capillaries and in necrotic muscle fibers, pointing toward inflammatory myopathy. IMNM was suspected histologically. Cerebrospinal fluid analysis was unremarkable.

She was empirically treated with intravenous cefuroxime for 7 days. During the 2-week hospitalization, her symptoms alleviated without any immunosuppressive therapy, she regained her normal ability to move, and her CRP declined to 6 mg/l. She was discharged from the hospital and referred to a rheumatologist because of suspected seronegative IMNM.

**Fig. 3:**
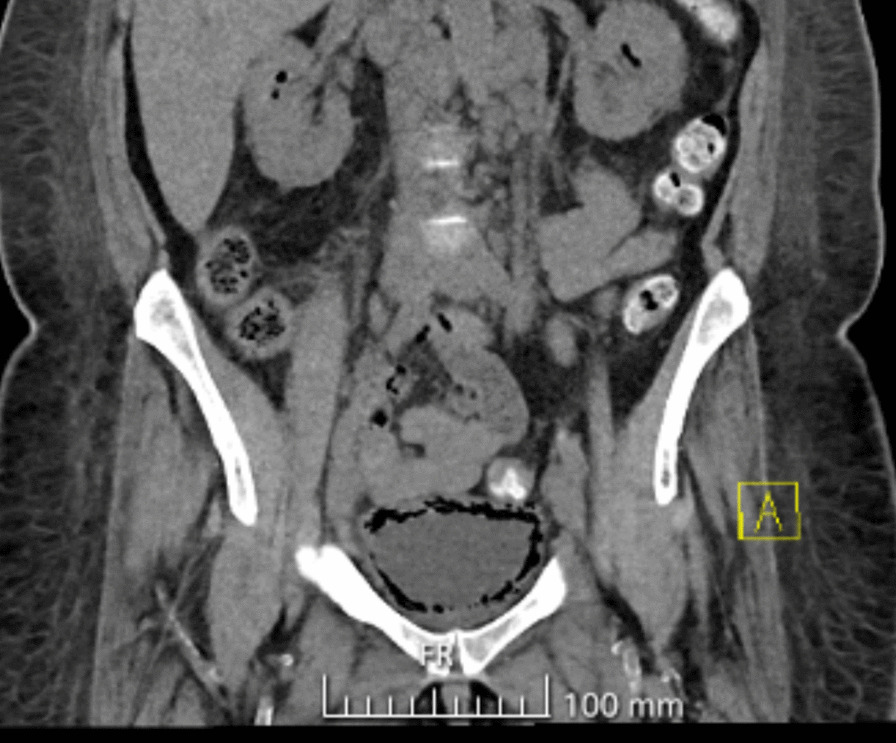
Emphysematous pyelitis and cystitis in the computed tomography of the abdomen

After a month at the rheumatology appointment, the patient presented with a new-onset 5-day history of marked swelling, weakness, and mild pain in her leg. Clinical examination showed reduced muscle strength in the right leg in hip flexion, knee extension and flexion, and ankle dorsiflexion, and marked edema in both legs. CRP level was 177 mg/l, and CK was only mildly increased to 357 IU/l. She was admitted to the hospital for a suspected infection and worsening necrotizing myopathy of unknown etiology. She had no fever and no urinary or abdominal symptoms. Again, urine sample showed pyuria, and urine culture was positive for *Escherichia coli*, sensitive to all studied antibiotics. Brain computed tomography (CT) showed no abnormalities that could explain the weakness of the right leg. A CT of the abdomen revealed emphysematous cystitis and pyelitis (Fig. 3), for which she was treated with intravenous ertapenem 0.5 g daily. Her blood cultures were negative.

Initially, pharmacological treatment was targeted toward IMNM and the infection concomitantly. She received oral corticosteroid treatment at a relatively low dose of prednisolone 20 mg/day because of the acute infection and atypical presentation. She received 2 days of intravenous immunoglobulin (total dose 2 g/kg), considering its beneficial safety profile regarding concomitant infection and insulin-dependent diabetes.

The charts of the first hospital admission were re-reviewed, and the initial presentation of muscle symptoms and findings were considered to be best explained by diabetic myonecrosis, although bilateral or multifocal involvement is uncommon [2]. The diagnosis of diabetic myonecrosis was supported by the existing risk factors for diabetic myonecrosis (poorly controlled diabetes, microvascular complications, and ESRD). The initial symptoms of muscle pain, stiffness, an edema with high CRP, and low CK levels were compatible with this condition [3]. The muscle biopsy was also re-evaluated and considered to be compatible with diabetic myonecrosis. At histological re-evaluation microvascular sclerosis was noted, supporting ischemic etiology of myonecrosis. Low-dose aspirin and analgesics were started as a treatment for the underlying diabetic myonecrosis, and prednisolone was stopped.

**Fig. 4:**
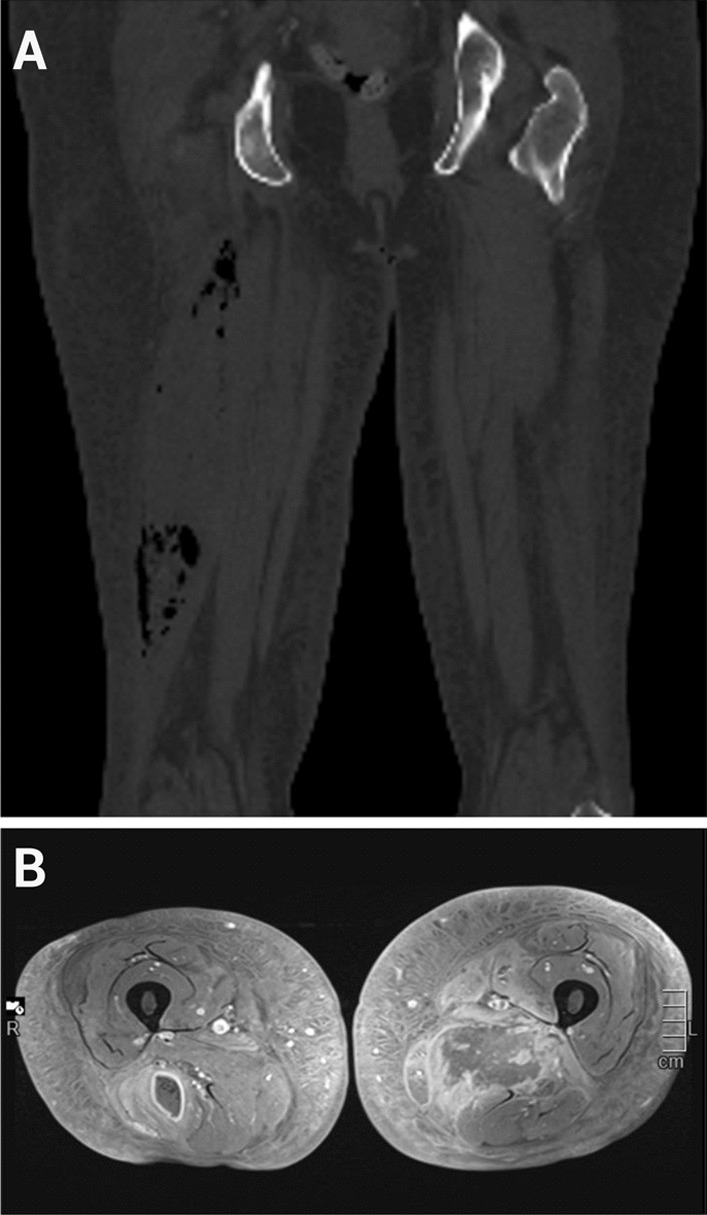
**A** Computed tomography of the thighs without contrast enhancement showing emphysematous myositis of the hamstring muscle in the right thigh. **B** Intravenous gadolinium-enhanced MRI scan of both thighs showing the emphysematous myositis and abscess with an enhancing ring and gas bubbles in the right biceps femoris muscle, and diabetic myonecrosis in the left adductor magnus muscle, with unenhancing intramuscular necrosis surrounded by enhancing peripheral reactive muscle edema

A few days after the second hospitalization, her symptoms worsened: her right hamstring became more painful, swollen, indurated, and markedly sore. Ultrasound of the right thigh showed marked edema and emphysema with no apparent abscess in the hamstring muscle. These findings were confirmed in a CT scan of her right thigh: marked edema and emphysema were detected in the caput longum of right biceps femoris and semimembranosus muscles (Fig. 4A). The distribution of the air in the muscles was not suggestive of necrotizing fasciitis. Because of rather mild and stable clinical symptoms, conservative treatment was reinforced by a consulting plastic surgeon. In a repeated CT scan of the thighs, an abscess of the right hamstring was observed (Fig. 4B). The drainage of the abscess produced 500 ml of pus, with alleviation of symptoms and a decrease in inflammatory markers. The culture of the pus was positive for *Escherichia coli*, sensitive to all studied antibiotics.

**Fig. 5:**
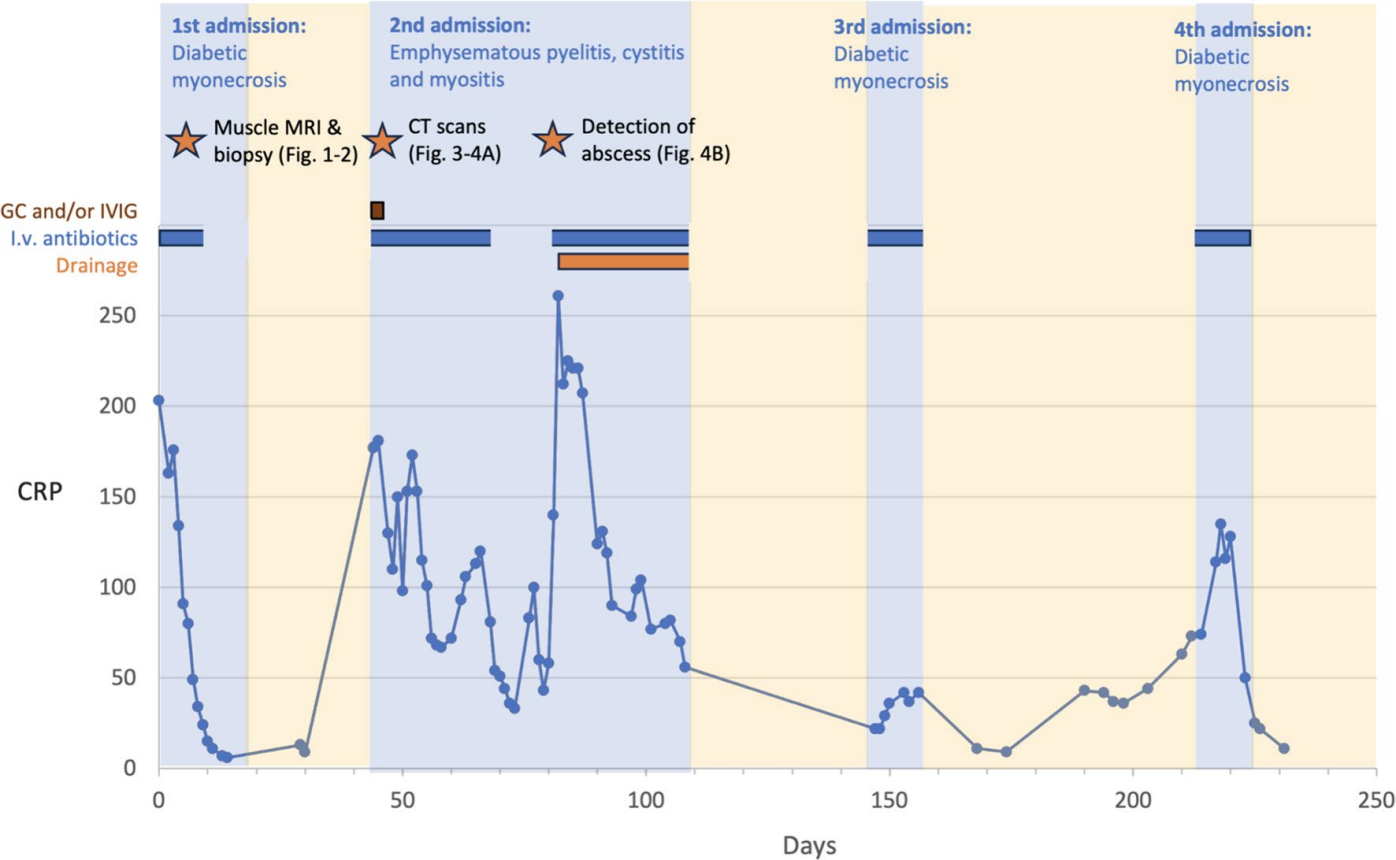
Timeline of the events

Intravenous ertapenem was continued for pyomyositis and muscle abscess for 3 weeks. Upon discontinuation of ertapenem, the patient complained of worsening of muscle pain in the left buttock and right hamstring area. An MRI of the pelvis and thighs showed increased edema and myonecrosis in the left abductor compartment and reoccurring compartmentalized abscesses of the right hamstring muscle. Antibiotic treatment with intravenous cefuroxime was reintroduced. The plastic surgeon was again in favor of conservative treatment, even though the abscess could not be drained because of small compartments. She recovered slowly with a 4-week course of intravenous cefuroxime and physical therapy, and was discharged with peroral cefalexin, which was continued until further notice as a preventive therapy. A timeline of given treatments is shown in Fig. 5.

She developed two more probable episodes of diabetic myonecrosis during the following months. First, she developed pain, swelling, and stiffness in her left antebrachial muscles, and the MRI of the elbow showed muscle and subcutaneous edema and possible muscle necrosis particularly in brachialis and brachioradialis muscles. Later, she had a milder episode of swelling and pain in the left distal quadriceps muscle. Both of these episodes resolved within a week.

## Discussion

We describe a case of diabetic myonecrosis complicated by emphysematous pyomyositis and an abscess caused by *E. coli*. Initially, the patient was suspected to have IMNM, another cause of necrotizing myopathy. Diabetic myonecrosis, pyomyositis, and IMNM share common features such as muscle pain, swelling and weakness, muscle edema in imaging, and muscle necrosis. However, many differences also exist in their clinical presentation, disease course, imaging, histology, laboratory results, and extramuscular symptoms (Table 1). The importance of prompt diagnosis is emphasized by completely different treatment approaches. In addition to these three entities, necrotizing myopathy can be caused by other myositides, muscle dystrophies, connective tissue diseases, drugs, viral infections, and graft-versus-host disease, which must be kept in mind in the differential diagnosis [1].

**Table 1:** Comparison of diabetic myonecrosis, pyomyositis, and immune-mediated necrotizing myopathy

	Diabetic myonecrosis	Pyomyositis	IMNM
Risk factors	Poorly controlled diabetes, especially type 1 diabetes; microvascular complications; and end-stage renal disease.	Pre-existing muscular abnormality from strain, trauma, parasite, ischemia, and so on. Immunocompromised state is a risk factor. More common in tropical areas.	Statin treatment for anti-HMGCR-positive IMNM, cancer for seronegative IMNM.
Clinical symptoms			
Muscle pain	+++	+++	+
Muscle swelling	+++	+++	−
Muscle weakness	+	+	+++
Constitutional symptoms	+	+++	+
Affected muscles	Usually a single muscle group and unilateral. Lower limbs more often affected, and thigh muscles more often than calves. Upper limb involvement possible. Bilateral involvement in 8.4% [2].	Usually a single muscle group and unilateral, and most often in lower limb muscles. Multiple lesions can be present. Diffuse in 10–20% of cases [13].	Proximal bilateral muscle weakness predominantly in the lower extremities, but often also upper limb involvement. Dysphagia may be present.
Extramuscular symptoms	Not present.	Not present.	Anti-SRP-antibodies increase the risk of myocarditis and interstitial lung disease. Skin and joint involvement common in many IIMs, but usually not in IMNM.
Onset of symptoms and clinical course	Sudden onset, self-limiting in 1–3 weeks. Reoccurrence in 47% [2].	Acute or subacute onset. Stage 1 “invasive phase”: 1–3 weeks of swelling, pain, and mild-moderate constitutional symptoms. Stage 2 “suppurative phase”: formation of an abscess. Stage 3 “late stage”: obvious local signs of an abscess, sepsis, and fever [7].	Onset within a few weeks or months, with a chronic and long-standing course.
Diagnostic tools			
CRP	Increased in 80%, mean CRP of 11 cases 153 mg/l [3].	Clearly elevated.	Slightly or moderately elevated.
Creatinine kinase	Normal in 50%, slightly elevated (< 1000) in 50% [3].	Normal or only minimally elevated [13].	High levels, typically 4000–8000 IU/l. Normal CK with normal muscle mass largely rules out IMNM [10].
Myositis-specific antibodies	Negative.	Negative.	Present in 80% (usually anti-SRP or anti-HMGCR autoantibodies), 20% seronegative [10].
Other laboratory tests		Intramuscular content culture needed. Blood cultures positive only in 5–35%	
Imaging (MRI)	Muscle edema, subcutaneous edema, unenhancing intramuscular necrosis surrounded by enhancing peripheral reactive muscle edema.	Muscle edema, hyperdense areas corresponding to intramuscular necrosis or collections, and sometimes definite abscess with enhancing peripheral ring.	Muscle edema, and in later stages, fatty replacement and muscle atrophy.
Emphysema present	No.	Possible.	No.
Muscle biopsy	Muscle necrosis, inflammatory cell infiltration, and sometimes muscle fiber regeneration and endomysial fibrosis.	Acute inflammation characterized by neutrophil-rich infiltrates.	Necrotic muscle fibers as the predominant abnormality, with different stages of necrosis and random distribution, and variable lymphocytic infiltrates with macrophages.
Treatment	Initial rest, analgesics, strict glycemic control, physiotherapy, and low-dose aspirin if not contraindicated.	Combination of percutaneous or open surgical drainage along with antimicrobial therapy guided by culture results.	Prednisolone, immunosuppressants such as methotrexate and rituximab, and intravenous immunoglobulins.

Diabetic myonecrosis, that is, muscle infarction, refers to spontaneous ischemic necrosis of skeletal muscle among people with diabetes [3]. The exact pathogenesis of diabetic myonecrosis remains unknown, but arteriosclerosis and diabetic microangiopathy are likely to play a role [2]. Many predisposing factors were present in our patient, including microvascular complications and end-stage renal disease [3]. The diagnosis of diabetic myonecrosis is based on a high level of suspicion in patients with diabetes and microvascular complications, and acute to subacute onset of swelling, pain, and tenderness of muscle(s) [4]. MRI is helpful in diagnosis, and muscle biopsy can be used in atypical cases to exclude other causes [4]. Although the involvement of only one muscle group is the most common presentation, bilateral involvement is possible [2]. The extent of our patient’s symptoms, that is, presence of edema and weakness in both the thighs and calves, is not typical for diabetic myonecrosis, but has been described before [5, 6]. This uncommon presentation may have contributed to the diagnostic delay and suspicion for IMNM in her case.

The patient had *E. coli* emphysematous pyelitis and cystitis, and the infection probably spread to the infarcted muscle via a hematogenous route. Pyomyositis often occurs in muscles with pre-existing pathologies [7], and the ischemic muscle could serve as a favorable breeding ground for the *E. coli*. Anaerobic conditions are likely to have induced the facultative anaerobe *Escherichia coli* to ferment glucose, producing carbon dioxide and hydrogen that may appear as gas on imaging [8]. To our knowledge, only one case of co-occurrence of emphysematous cystitis and myositis has been reported before, also in a diabetic patient and caused by *E. coli* [9].

The most common pathogen in pyomyositis is *Staphylococcus aureus*, but also other gram-positive, gram-negative, and anaerobic bacteria such as group A *Streptococcus*, *Escherichia coli*, *Klebsiella pneumoniae*, and *Clostridioides* can be causative [7]. The clinical course of pyomyositis has been described as follows [7]: an “invasive stage” of usually 10–21 days with onset of dull muscle pain, low-grade fever, and localized edema, without marked muscular tenderness; a “suppurative stage,” with fever, abscess formation with more evident muscular mass/swelling, and tenderness in affected muscles; and a “late stage” with severe pain, high fever, sepsis, and multiorgan dysfunction with high mortality. The first two stages can be identified also in our patient’s history.

The patient was initially suspected to have IMNM. However, rather low CK levels and very high CRP levels were not compatible with this condition, as IMNM patients typically have only slightly or moderately elevated CRP, and typical CK values rise to several thousand IU/l (Table 1). Furthermore, she did not have myositis-associated or myositis-specific antibodies that are present in 80% of patients with IMNM [10]. Moreover, her symptoms resolved spontaneously during 2 weeks, which led us to consider alternative diagnoses. However, the MRI and biopsy findings could have been compatible with seronegative IMNM. In the muscle biopsy, we detected immunohistochemical expression of complement C5b-9 MAC in capillaries and necrotic myofibers. The expression of C5b-9 MAC is used as diagnostic support in dermatomyositis as well as in other immune-mediated inflammatory myopathies [11]. However, the capillary C5b-9 MAC reactivity has been shown to be present also in non-inflammatory muscle biopsies of diabetic patients with microvascular sclerosis [12].

Diabetic myonecrosis reoccurs in 40–50%, mostly in another muscle group within 6 months of the initial diagnosis, as happened also in our patient [2, 3]. We also considered the possibility that the reoccurrence was caused by re-infection, but diabetic myonecrosis was deemed more likely, given the milder course and the involvement of new muscle groups. Of note, the possibility of carnitine palmitoyltransferase II deficiency, and autosomal recessive disorder and a cause of recurrent rhabdomyolysis, was considered, but no genetic testing was done due to the patient’s low CK levels and high age of onset.

The main strength in the management of this case was the continuous collaboration between doctors from internal medicine subspecialties, radiologists, and pathologists.

An additional learning point is that, against all odds, our case presented with two co-occurring rare muscle diseases. Neither diabetic myonecrosis nor pyomyositis explained all clinical symptoms and findings alone. In cases of atypical clinical presentation, misdiagnosis is always a reasonable concern, but two or more coexisting pathologies can also cause unusual clinical patterns.

## Conclusion

Identifying the etiology of necrotizing myopathy may be challenging and requires a multidisciplinary assessment of internists, pathologists, and radiologists. Moreover, the presence of two rare conditions concomitantly is possible in cases with atypical features.

## Data Availability

All data underlying the results are available as part of the article, and no additional source data are required.

## Abbreviations

CD: Cluster of differentiation
CK: Creatinine kinase
CRP: C-reactive protein
CT: Computed tomography
ESRD: End-stage renal disease
HMGCR: 3-Hydroxy-3-methylglutaryl-coenzyme A reductase
IMNM: Immune-mediated necrotizing myopathy
MAC: Membrane attack complex
MHC: Major histocompatibility complex
MRI: Magnetic resonance imaging
SRP: Signal recognition particle
